# How Nurses Assess Interventions to Promote Patients Sleep on Hospital Wards: Results of a Self‐Report Survey

**DOI:** 10.1111/scs.70329

**Published:** 2026-10-25

**Authors:** Miia Vasse, Elina Paala, Mari Kangasniemi, Marita Koivunen

**Affiliations:** ^1^ HUS Helsinki University Hospital, University of Helsinki and Helsinki University Hospital Helsinki Finland; ^2^ Wellbeing Services County of Satakunta Pori Finland; ^3^ Department of Nursing Science University of Turku Turku Finland

**Keywords:** cross‐sectional study, hospital, inpatient, nursing, sleep promotion

## Abstract

**Aim:**

The aim of this study is to describe how nurses assess interventions to promote patients' sleep on hospital wards.

**Desing:**

The study was a cross‐sectional survey, and it was conducted among nurses in Finnish specialist healthcare in 2022.

**Methods:**

Out of 482 eligible participants in the sampling frame, 129 nurses returned a completed questionnaire. The open‐ended question was answered by 36 participants. Data were analysed using statistical methods and inductive content analysis.

**Results:**

In general, participants assessed that they perform well in sleep‐promoting activities in a hospital ward. Participants gave the highest assessment to supporting patients' mental and emotional well‐being. Minimising disturbance related to nursing interventions got the lowest scores. Based on the open‐ended questions, respondents described that the realisation of patients' sleep support in the hospital related to the care environment, patient placement within the ward, resources available for nursing interventions, sleep‐supporting aids, care culture and training related to supporting patients' sleep.

**Conclusion:**

Nurses assess that they are implementing patient sleep‐promotion interventions well. Nurses realise patients' sleep promotion as a part of their daily activities. Nurses can promote patient's sleep with small activities, as long as the organisation takes care of the nurses' skills and ensures practical interventions so that the patient gets enough rest during the treatment period.

## 1 Introduction

Good‐quality sleep is the foundation of health and consists of adequate duration, proper timing, and a lack of disturbances. A healthy adult needs 7–9 h of sleep per night, and the need for sleep increases during illness [1]. Sleep has a comprehensive effect on vital functions such as the metabolic, cardiovascular, neurologic, and endocrine systems, as well as psychological well‐being and cognitive performance [2]. Prolonged sleep deprivation is associated with obesity, diabetes, hypertension, heart disease, stroke and depression. Even a short‐term lack of sleep can increase pain and impair immune function [1].

In a hospital, sleep quality can significantly slow down and complicate a patient's recovery on several levels. Patients may suffer from short sleep periods and awakenings at night. Sleep can be disrupted because of the patient, or due to ward‐related reasons [3, 4, 5, 6]. In terms of patient‐related reasons, the patient may suffer from untreated pain and other symptoms, or they may be worrying about illness and not having an opportunity to be involved in their treatment [4, 5, 7]. Sleep and pain are closely linked; sleep deprivation is proven to lower the pain threshold. This means patients experience pain more intensely and may require more pain medication [8]. Sleep is critical for tissue repair and wound healing [9]. In addition, sleep deprivation is a major risk factor for delirium, and it also impairs memory, concentration and decision‐making. Lack of sleep lowers mood, causes irritability, and can make emotional regulation difficult. It is also linked to increased experiences of exhaustion [10]. Ward‐related reasons refer to care routines and disturbing lighting and noises on the ward [3, 4, 6]. In addition, an uncomfortable bed, pillow, or sleeping position can disturb sleep [3, 5].

It has been found that sleep deprivation can extend hospital stays by 1–8 days [9]. Poor sleep patterns established in the hospital can persist for months after discharge, hindering the return to normal functioning and increasing the risk of readmission [11]. Optimal patient sleep on hospital wards is relevant and topical from a healthcare perspective. In EU countries, the average length of stay for inpatient care in acute hospitals is 4–9 days, and in Finland, it is 6 days [12]. Currently, the Finnish healthcare system is undergoing a significant structural change, one of the aims being to increase the number of outpatient services compared with hospital care [13, 14]. The goal is to keep hospital stays as short as possible, so the importance of good‐quality sleep for the patient is increasing. As the length of stay decreases, it becomes even more important to promote the patient's sleep during the stay.

From a nursing perspective, it is essential to recognise that supporting a patient's sleep occurs within the care relationship and is related to both the personality of the individuals and the context in which the patient is being cared for. The quality of care depends not only on clinical tasks but also on interpersonal relationships and the organisational context in which care is delivered [15]. To promote the nursing relationship and the quality of care, there are interventions that ensure uniform practices among nurses, while taking into account the patients' individual needs [16]. Sleep promotion is the core nursing intervention in the hospital [17] in terms of maintaining patients' good health and recovery from illnesses [3, 18]. Promoting patients' sleep and rest in a hospital setting is a fundamental dimension of high‐quality, evidence‐based nursing. Within international nursing frameworks, sleep is systematically conceptualised as a core physiological necessity rather than merely a component of comfort. For instance, the Fundamentals of Care (FoC) framework categorises rest and sleep as an essential physical requirement that directly influences patient recovery, clinical outcomes, and holistic well‐being. The FoC framework focuses on practical everyday nursing actions, which help the patient manage their basic needs. Supporting sleep and rest is an important part of meeting the patient's unique care needs [19]. A recent literature review confirms that promoting sleep hygiene is an integral part of holistic nursing care [20]. As part of fundamental care, nurses support the patient's sleep through practical interventions that eliminate sleep‐disrupting factors and create conditions conducive to falling asleep and getting good sleep in the hospital. This is important because good sleep promotes the patient's recovery, the healing of tissue damage, and mental well‐being [20, 21]. Simple fundamental care interventions have a significantly positive impact on sleep quality [22].

Sleep‐promoting interventions refer to the activities nurses undertake to facilitate optimal sleep for their patients [23]. Patients' sleep can be promoted by paying attention to the environment, nursing scheduling, emotional support, patient needs, and medication. In terms of the environment, patients' sleep can be promoted on the ward by adjusting the lighting, ensuring the right temperature, creating a calm atmosphere, minimising noise, and helping the patient find a comfortable sleeping position [18, 23, 24, 25] Clustering care in the ward helps decrease the number of awakenings and promotes sleep [23]. Caring conversations with patients can calm them, reduce anxiety, and promote patients' sleep [26, 27]. When nurses take care of patients' physical needs, such as taking them to the toilet before going to bed, they promote the patients' sleep as well. Encouraging patients to be active during the daytime improves their sleep at night [17, 23] Sleep can be promoted by using medication. Medication can relieve symptoms, as in the case of painkillers, or it can be sedative or sleep medication [26]. Sleep promotion is a basic clinical need, and patients' individual needs for interventions should be considered [28].

Nurses are often aware of sleep‐disturbing factors. According to previous studies, nurses have a knowledge deficit regarding sleep‐promoting interventions [17]. Previous studies on patients' sleep in the hospital have mostly focused on sleep‐disturbing factors and the effect of a certain sleep‐promoting intervention [e.g., 3–6]. Research has often been carried out from the patients' perspective (e.g., 3, 4, 7 [29, 30, 31]). There is a knowledge gap in understanding how nurses assess their own activities in promoting patients' sleep in hospital wards [32, 33, 34]. Nurses assessing their own activities can enhance understanding of nurses' knowledge and abilities when promoting sleep.

## 2 Materials and Methods

### 2.1 Aim

The aim of the study is to describe how nurses assess interventions to promote patients' sleep on the hospital ward. The overall goal is to produce knowledge that nurse managers can use to support the daily activities of nurses in hospital wards and thus ensure the quality of fundamental patient care.

Research questions are as follows:

How are nursing interventions that promote patients' sleep realised as assessed by nursing staff?

What factors contribute to the realisation of nursing interventions related to patients' sleep promotion?

### 2.2 Study Design and Setting

This was a cross‐sectional survey study of nurses' self‐assessment, conducted in Finnish specialised healthcare in 2022. Data were analysed using statistical methods and inductive content analysis. The study involved nursing staff in two central hospitals (Hospital A and Hospital B) working in adult inpatient somatic wards. A somatic ward is a hospital ward used to treat physical or bodily diseases and injuries. The hospitals are typical Finnish central hospitals for specialised healthcare and are nearly the same size in terms of patients' bed‐days. In these hospitals, the average bed‐days for these types of somatic wards are 3–4 days.

### 2.3 Data Collection

The data were collected using a questionnaire that was developed for this study. The structure of the questionnaire is based on a nursing protocol for patients' sleep in the participating organisation. The organisation developed the protocol using previous literature, patient interviews, and workshops for nurses [35]. In this study, we formulated 64 items based on the protocol as a pool for the first version of the questionnaire. After that, we removed overlapping items and revised wording in the items [36], resulting finally in 48 items organised into six sub‐scales. The sub‐scales were as follows: (1) pain and other symptom management (eight items); minimising disturbance related (2) to nursing interventions (ten items); and (3) to round‐the‐clock activities on the ward (eight items); (4) providing a good sleeping environment in the patient room (eight items); (5) paying attention to fear, worry, uncertainty, and confusion (seven items); and (6) promoting the maintenance of a healthy circadian rhythm (seven items). The respondents were asked to assess their own performance in patient sleep promotion on a Likert‐type scale where 1 = poorly realised, 2 = satisfactorily realised, 3 = moderately realised, 4 = well realised, 5 = excellently realised, and 6 = does not apply to me. At the end of the questionnaire, there was an open‐ended question where participants were asked to describe what issues or factors would contribute to the realisation of nursing interventions promoting the patient's sleep. In addition, we collected background characteristics including organisation, occupational title, last degree, work experience in healthcare, work experience in the current ward, type of shift work, and participation in training related to sleep promotion in the last 2 years.

Sampling frame (*N* = 482) consisted of the total nursing staff working on the wards in Hospitals A and B. The sample included all nurses working in the adult somatic wards of those hospitals. The data were collected in autumn 2022. The contact persons in the study organisations distributed the questionnaires to eligible staff members via email. The response period was 4 weeks, and a reminder email was sent during the data collection period.

### 2.4 Data Analysis

Quantitative data were analysed by statistics using IBM SPSS Statistics for Macintosh versions 28 and 29, and qualitative data were analysed using inductive content analysis. Frequencies and percentages were used to describe the quantitative background data. Six mean sum variables were formed from the scales of the instrument. Means, medians, minimums and maximums and standard deviations were calculated for the six sum variables. The effects of background variables on the items were examined using cross‐tabulation and the Kruskal–Wallis test (*p* = 0.05). The impact of background variables on the realisation of the items was analysed based on these results using the chi‐squared test or Fisher's exact test, depending on the number of missing cells, as well as the Mann–Whitney *U* test.

The open‐ended question in the survey was analysed using inductive content analysis. The responses were carefully read, sentences were condensed, and meaningful words or phrases relevant to the study were identified and coded. Subsequently, themes were formed from the congruent codes [37]. The data were formed into five themes related to the care environment, patient placement within the ward, factors related to resources, sleep‐promoting aids, and care culture and training related to promoting patient sleep.

### 2.5 Ethical Considerations

This study received ethical approval from the ethics committee of the Higher Education Institutions (Ethics Committee for the Human Sciences at Satakunta Higher Education Institutions, decision dated 10 January 2022). Permission to conduct the study was obtained from the hospital authorities (Decisions: Satakunta Hospital District, 2 February 2022; Hospital District of Central Finland, 7 February 2022). The questionnaire was accompanied by an introductory letter with information about the aim of the study and assurance of anonymity and confidentiality. Responding to the survey was considered informed consent to participate in the research. Privacy, confidentiality, and anonymity were ensured throughout the process [38, 39, 40].

## 3 Results

Out of 482 eligible participants in the sampling frame, 129 returned a completed questionnaire (total response rate of 27%). The open‐ended question was answered by 36 participants. More than two‐thirds of the respondents (78%) worked in Hospital A. Most of the respondents were registered nurses (93%) and worked three shifts (90%). Only 12% of the respondents had received training related to patient sleep promotion in the past 2 years (Table 1).

**TABLE 1:** The background characteristics.

	*n* (%)
Hospital	
Hospital A	101 (78)
Hospital B	28 (22)
Occupational position	
Registered nurse, RN	119 (93)
Practical Nurse	7 (6)
Other	2 (1)
Last degree	
Registered nurse, RN, bachelor of health care	119 (93)
Registered nurse, RN, master of health care	2 (1)
Master of nursing science	1 (1)
Practical Nurse	7 (5)
Work experience in healthcare	
0–3 years	17 (13)
4–6 years	20 (16)
7–15 years	34 (26)
Over 15 years	57 (45)
Work experience in the current ward	
Under 1 year	20 (16)
1–3 years	33 (25)
4–6 years	20 (16)
7–15 years	27 (21)
Over 15 years	29 (22)
Type of shift work	
daytime work	0 (0)
two‐shift work	10 (8)
three‐shift work	117 (90)
night work	2 (2)
Sleep‐promotion‐related training in the past 2 years	
Yes	16 (12)
No	113 (88)

The respondents assessed that they perform well in all areas related to promoting patient sleep, with an overall average score of 4.14 for all scales. They estimated that the issues they take into account best (mean 4.24) are the patient's fears, concerns, anxiety, and confusion, while participants rated minimisation of nursing‐related interventions the weakest (mean 3.99) (Table 2).

**TABLE 2:** The findings of the sub‐areas of the nursing interventions to promote patients' sleep.

	Mean	Md	SD	Min	Max
Paying attention to fear, worry, uncertainty and confusion	4.24	4.29	0.533	2.14	5.00
Minimising disturbance related to round‐the‐clock activities on the ward	4.20	4.25	0.607	2.38	5.00
Promoting the maintenance of a healthy circadian rhythm	4.18	4.14	0.584	2.86	5.00
Pain and other symptom management	4.16	4.25	0.530	2.25	5.00
Providing a good sleeping environment in the patient room	4.09	4.19	0.619	2.50	5.00
Minimising disturbance related to nursing interventions	3.99	4.00	0.499	2.70	5.00

The item‐specific means (M), median (Md), standard deviation (SD), minimum (Min) and maximum (Max) are described in Table 3.

**TABLE 3:** Participants' assessments for using sleep promoting interventions.

	*n*	Mean	Md	SD	Min/Max
**Paying attention to fear, worry, uncertainty and confusion**					
I provide medication for falling asleep as needed	122	4.58	5	0.558	3/5
I request a discussion with a doctor if the patient so desires	122	4.34	4	0.688	2/5
I ensure the patient receives timely and sufficient information	124	4.25	4	0.717	2/5
I ensure that the patient feels heard and understood	125	4.24	4	0.677	2/5
I ensure that patients feel safe and informed	125	4.22	4	0.694	2/5
I take psychological factors into account and offer additional support	126	4.07	4	0.792	2/5
I encourage patients to talk about their mental health issues during the day	127	4.00	4	0.766	2/5
**Minimising disturbance related to round‐the‐clock activities on the ward**					
I operate quietly at night and avoid tasks that create noise	120	4.57	5	0.632	2/5
I respond quietly to patient calls during the night	119	4.39	5	0.856	1/5
I offer earplugs to promote sleep	126	4.33	4	0.810	1/5
I keep patient room doors closed	125	4.30	5	0.815	2/5
I perform only essential care tasks for emergency patients	108	4.27	4	0.756	2/5
I keep pathways clear	123	4.24	4	0.761	2/5
I admit patients to the ward quietly at night	115	3.77	4	1.035	1/5
I report any malfunctions I notice	128	3.75	4	1.004	1/5
**Promoting the maintenance of a healthy circadian rhythm**					
I take care of the lighting in the patient room	125	4.58	5	0.638	2/5
I encourage the patient to be active during the day	127	4.43	5	0.729	2/5
I ensure the patient's ability to get out of bed	126	4.38	4	0.631	3/5
I enable reading and watching TV during the day	127	4.22	4	0.872	1/5
I encourage family members to stay in touch with the patient	128	4.06	4	0.911	2/5
I calm down activities in patient rooms in the evenings	127	3.82	4	0.895	1/5
I gently wake up patients who are sleeping during the day	127	3.79	4	1.005	1/5
**Pain and other symptom management**					
I ensure individualised positioning therapy	123	4.50	5	0.645	2/5
I encourage the patient to describe their symptoms	127	4.46	5	0.774	1/5
I inquire about pain before bedtime	125	4.43	5	0.699	1/5
I ensure adequate symptom‐relieving medication	127	4.40	4	0.621	3/5
I medicate symptoms before bedtime	126	4.40	4	0.633	2/5
I alleviate pain through non‐pharmacological means	124	3.92	4	1.001	1/5
I proactively ensure adequate pain medication at night	125	3.90	4	0.896	1/5
I use a pain scale to assess pain	126	3.25	3	1.136	1/5
**Providing a good sleeping environment in the patient room**					
I provide patients with additional clothing, blankets and pillows	125	4.59	5	0.569	3/5
I close window blinds at night	125	4.50	5	0.747	1/5
I medicate restless patients as needed	123	4.47	5	0.605	2/5
I ensure windows are closed at night	100	4.32	5	0.973	1/5
I relocate a disruptive patient from a large patient room	110	4.19	4	0.924	1/5
I ensure that visits do not disturb patients' rest	126	3.74	4	1.021	1/5
I direct patients to the day room for reading or watching TV	104	3.56	4	1.205	1/5
I ensure fresh air in patient rooms	120	3.36	3.5	1.187	1/5
**Minimising disturbance related to nursing interventions**					
I use dim lighting during the night	119	4.64	5	0.565	2/5
I only perform essential care tasks during the night	119	4.50	5	0.623	2/5
I conduct nighttime measurements discreetly	121	4.35	5	0.793	1/5
I consolidate care tasks to be performed simultaneously	126	4.30	4	0.684	2/5
I shield other patients from light	119	4.27	4	0.831	1/5
I time medication administration to avoid waking the patient	122	4.24	4	0.882	1/5
I alleviate thirst for patients who cannot eat	120	4.12	4	0.724	2/5
I consider patients' need for rest in all activities	128	4.00	4	0.753	2/5
I replace poorly functioning IV lines in the evening	116	3.89	4	0.976	1/5
I provide eye masks	114	1.68	1	1.043	1/5

### 3.1 Participants' Assessments for Using Sleep‐Promoting Interventions

#### 3.1.1 Paying Attention to the Patient's Fear, Worry, Uncertainty, and Confusion

Based on nurses' self‐assessment, the best‐implemented interventions focused on paying attention to the patient's fear, worry, uncertainty, and confusion. Each item of this area was performed well, with little variation. We found three statistically significant results in this section. Nurses with more than 15 years' work experience in the healthcare sector performed medication care statistically significantly (*p* = 0.04) less well (mean 4.36) compared to nurses with less work experience. When comparing hospitals, there was a statistically significant difference (*p* = 0.03) between nurses who requested a doctor to discuss treatment‐related uncertainties with the patient (Hospital A mean 4.67 and Hospital B mean 4.23). Participation in training related to supporting patient sleep had statistical significance in this scale, as three items were performed better according to those who did not participate in the training.

#### 3.1.2 Interventions Related to the Hospital Environment and Round‐The‐Clock Activities

Based on the participants' responses, interventions related to the hospital environment and round‐the‐clock activities on the ward were performed well, except for actions related to reporting malfunctions and receiving emergency patients, where performance was rated as moderate. Statistically significant differences (*p* = 0.04) between hospitals were observed only when nurses assessed keeping patient room doors closed and walking quietly through them, with Hospital B having a mean score of 4.40 and Hospital A a mean score of 3.90. Participation in training related to supporting patient sleep was statistically significant (*p* = 0.045) only for one item. Those who participated in the training performed poorly (mean 3.9) in providing only essential care and discussions when transferring emergency patients to a large room compared to those who did not participate in the training (mean 4.3).

#### 3.1.3 Promoting a Healthy Circadian Rhythm for Patients

Respondents assessed that they performed well (mean 4.18) in supporting a healthy circadian rhythm for patients, with only two items being performed moderately. Hospital‐specific statistically significant differences (*p* = 0.03) were observed in nurses enabling patients to read and watch TV during the daytime (Hospital A mean 4.4 and Hospital B mean 4.17). The respondents' participation in training related to supporting patient sleep was found to be statistically significant for three items, with all three items showing that those who participated in the training performed these actions less effectively than those who did not participate in the training.

#### 3.1.4 Managing Pain and Other Symptoms

The respondents reported that overall, they pay attention to and manage pain and other symptoms of illness well (mean 4.16). There was some variation between items. Respondents performed best (mean 4.5) in individual positioning therapy, while they performed functions related to pain and its assessment moderately. There was variation between hospitals in the use of pain scales (*p* = 0.01), as the use of pain scales was better performed in Hospital A (mean 3.7) compared to Hospital B (mean 3.1); however, there was no statistically significant difference between hospitals for other items. There was statistical significance (*p* = 0.02) in terms of work experience in the healthcare sector regarding performing individual positioning therapy. The impact of whether respondents had received training related to supporting sleep was found to affect one item (*p* = 0.02). Respondents who had received training were less likely to encourage patients to report their symptoms at all times of the day (mean 4.13) compared to those who had not received training (mean 4.5).

#### 3.1.5 Providing a Good Sleeping Environment

Nurses assessed that they performed well in actions related to providing a good sleeping environment, as all items were either well or moderately performed. Statistically significant hospital‐specific differences were observed in four statements within this scale, with all items being better performed in Hospital B. Participation in training related to supporting patient sleep was statistically significant only for the last item (*p* = 0.03). Those who had recevied training performed poorly in closing windows at night (mean 4.06) compared to respondents who did not receive training (mean 4.7). Work experience in the healthcare sector had statistical significance (*p* = < 0.01) only for the statement regarding additional clothing and blankets, as respondents with less than 4 years of work experience provided additional blankets less effectively than those with longer work experience.

#### 3.1.6 Managing Discomfort Related to Nursing Intervention

The management of discomfort related to nursing interventions was assessed to be the weakest in all scales, with an average score of 3.99. There was considerable variation within this area. Respondents assessed providing eye protection to be realised poorly (1.68). There was also variation between hospitals in the implementation of this item (*p* = 0.03), with Hospital A having an average score of 2.08, while Hospital B had an average score was 1.57. Hospital‐specific differences (*p* = < 0.01) were observed in the timing of patient medication, with Hospital A having a mean score of 4.07 and Hospital B a mean score of 3.87.

### 3.2 Participants' Description of Factors Contributing to Realisation of Patients' Sleep in the Hospital

Based on the open‐ended questions, respondents described that the realisation of patients' sleep in the hospital as being related to the care environment, patient placement within the ward, resources available for nursing interventions, sleep‐supporting aids, care culture and training related to supporting patient sleep.

#### 3.2.1 The Meaning of the Care Environment for a Patient's Sleep

Respondents felt that patients' sleep in the hospital could be improved by considering the proper care environment, including the type of patient room, air and lighting, and adaptability of rooms. Patient rooms were considered to be impractical. They were cramped, and items or patient beds had to be moved at night when a patient needed to go to the bathroom. This caused noise, which made it difficult for other patients to sleep. Additionally, air that was too cold or too hot was reported as a factor affecting sleep, especially when the air conditioning did not work properly or when windows could not be opened or had drafts. Proper air conditioning or functioning ventilation was also desired to eliminate unpleasant odours. Regarding lighting, nurses described motion‐sensor lights that turned on at full brightness at night, illuminating the entire patient room. Blinds should also function properly to keep the room dark enough, especially during summer or proper blackout curtains should be installed. Concerning beds, respondents mentioned that beds should not beep when used, and creaky beds should be fixed. Various sounds from door hinges, trash can lids and automatic doors opening were also described as noisy and respondents hoped that these would receive more attention and corrective actions.

#### 3.2.2 Patient Placement Within the Ward

Respondents described patient placement within the ward is also related to the realisation of patients' sleep in the ward. They described how, in multi‐patient rooms, the noises either made by other patients or related to their care disturbed patients' sleep. There may also be patients in the rooms who are restless or speak loudly and cannot be moved to a smaller room. Respondents expressed a desire for patients to be cared for in single or at most double rooms, which would also make it easier to realise care practices that support sleep. When this is not possible due to hospital facilities, respondents described attempting to pre‐emptively arrange patient placements to ensure the calmest room possible for all patients. However, this is not always feasible, as patients also arrive during emergency hours, and the level of care needed for patients can change quickly.

#### 3.2.3 Available Resources for Nursing Interventions

Staffing‐related resources available for nursing interventions were also identified as a factor influencing the realisation of patients' sleep. Respondents described that because of high nurse–patient ratios, they do not have enough time to carry out sleep‐promoting nursing activities, even if they want to. Respondents wished for improved resources so that each nurse would have fewer patients under their care, allowing for better planning and implementation of sleep‐supporting actions. They also expressed a desire for sufficient time to support patients' sleep and to have more time to talk with patients, which was described as being connected to promoting the patients' sleep.

#### 3.2.4 The Use of Sleep‐Supporting Aids

Realisation of patients' sleep also required sleep‐supporting aids such as eye masks and earplugs, but they are not always available. Respondents hoped that purchases of sleep‐promoting aids would be made so that the implementation of sleep‐supporting measures would be possible in these respects as well.

#### 3.2.5 Care Culture and Training

Respondents described that realisation of patients' sleep can be supported by developing a care culture and education related to supporting patient sleep. Respondents described how units should critically examine their operations and, if necessary, organise their activities in a way that would facilitate the provision of high‐quality care. This was seen as both an administrative issue, in terms of measures such as ensuring sufficient resources, and a cultural issue related to care practices, such as standardising procedures, as well as an individual nurses issue, in terms of acting as quietly as possible or timing medication correctly at nighttime, for example. Open discussion within the unit regarding supporting patients' sleep was desired, as was related training.

## 4 Discussion

Based on this study, nurses assess that, in general, they perform well in sleep‐promoting activities in the hospital ward. Nurses assessed that they performed best when supporting patients' sleep with regard to mental and emotional well‐being. Minimising disturbances related to nursing interventions got the lowest scores based on nurses' assessments. The results are in line with previous studies [e.g., 17, 26]. However, previous studies have indicated discrepancies with our results [5]: patients have reported that nurses do not sufficiently consider their emotional well‐being when supporting sleep [7, 24]. The reason might be that nurses lack knowledge regarding supporting patients' sleep realisation and therefore overestimate their own activities [5]. It is also possible that patients do not bring forth their needs for emotional support. This demonstrates the importance of good communication and a good relationship between patients and nurses, even when promoting sleep. Good communication supports patients in feeling safe, which helps them relax and tell nurses about their worries [7]. This also highlights the importance of understanding the patients' experience, and that it may differ from the understanding nurses have of a certain situation. It is also possible that when evaluating their own performance in supporting a patient's sleep, nurses want to protect their own professional skills. Nurses want to feel that they are doing their job well. If a nurse feels that they have done everything they can with the resources available, they will evaluate their own performance as successful, even if the end result, in this case, is poor sleep for the patient. Nurses may experience moral distress in situations where, for instance, due to a lack of resources, they are unable to act in the patient's best interest [41]. In order to objectively identify the quality of a patient's sleep and the implementation of related nursing interventions, a clinical observational study is needed in the future, in which both patient's sleep and the nursing activities can be observed.

According to previous research, using eye masks effectively promotes patients' sleep in the hospital [31]. In this study, nurses assessed giving eye masks to patients with the lowest scores, which lowers the scores of the whole sub‐area. In responses to the open‐ended questions, nurses described that eye masks were not available in the wards, which explains the low score. However, using eye masks would be an effective, relatively cheap way to promote patients' sleep. This is a good example of how nurse managers have an important role in sleep promotion by enabling proper resources and providing clear guidelines [17]. Clear sleep‐promotion guidelines could help nurses pay attention to factors related to sleep, even during the daytime. Nurse managers have an important role in creating a more evidence‐based culture and providing evidence‐based guidelines for sleep promotion [42].

In this study, the nurses assessed that they realised medication care well in terms of aiding the patients' sleep. They assessed that they were aware of possible sleep disturbance symptoms, and that pain management was adequate. On the other hand, nurses reported that they did not use pain scales to assess pain levels, nor did they use non‐pharmacological methods to alleviate pain. However, pain scales have been found to be an effective and reliable method for evaluating pain [26, 32] and useful in promoting patients' sleep. Clear guidelines on using pain scales could help in assessing pain and enhancing pain treatment, even when using non‐pharmacological means [26].

In our study, just one‐tenth of participants reported having further education on sleep promotion. This is in line with earlier studies showing that nurses lack education on sleep and how to promote it. According to previous studies, nurses lack knowledge regarding sleep promotion [17]. Promoting patients' sleep is not a specific action, but a process starting in the morning and needing to be considered at all times of the day. It is a part of comprehensive care and integrated into individualised care planning. Nursing interventions to promote patients' sleep in the hospital are part of high‐quality fundamental care [19]. Nurses need more knowledge about patients' sleep and its effect on patients' well‐being and recovery so they can pay attention to sleep‐promoting practices, even during the daytime. Nurses' sleep knowledge tends to be based on their own experiences rather than formal training. They are aware of the importance of sleep, but they have competing priorities of care and they also lack support from healthcare managers [23]. Nurses use their critical thinking, clinical judgement, and experience to determine the best course of action without needing permission for every task within their scope of practice [43]. Even in this case, a lack of education can lead to nurses supporting sleep based on their own experiences rather than evidence. Based on previous research it is known that due to a lack of knowledge, time and support from management, nurses support sleep based on their own experience and do not use evidence‐based interventions. It is also unrealistic to expect individual nurses to search for evidence‐based interventions to support patients' sleep [42]. Therefore, nursing managers have an important role in promoting the development of evidence‐based guidelines for sleep promotion. The successful implementation of sleep‐promoting practices requires active functions from nursing management as well as the strengthening of nurses' competence [44]. It is likely that in the future, length of stay in hospital wards will become shorter. For this reason, it becomes even more important to enhance nurses' skills and knowledge regarding sleep promotion.

Sleep promotion is often not a single action but a result of several nursing actions done during the day after considering the patients' individual needs. Ensuring the quality of a patient's sleep is part of the fundamental care provided. Our study confirms that simple fundamental care interventions have a significantly positive impact on sleep quality. When considering the promotion of a patient's sleep as part of holistic care, the aim is to address fundamental human needs, and sufficient sleep is one of these needs [19]. From this perspective, supporting sleep entails nurses building an interactive relationship with a patient, integrating the physical, psychosocial, and relational dimensions of care, and taking into account situational factors. If the ward culture is capable of integrating all these aspects to support the patient's sleep, it means the ward already possesses a high level of expertise and commitment to deliver comprehensive fundamental care. When the ward's operational culture enables the consideration and promotion of patients' sleep quality, it is likely that the ward also succeeds more broadly in delivering high‐quality holistic care. However, further research is needed on promoting patient sleep as part of the culture of fundamental care.

### 4.1 Strengths and Limitations

The limitations of this study are related to the questionnaire used and the recruitment of participants. Because there were no previous instruments from previous studies, we developed the questionnaire for this study. We integrated the nursing staff to participate in the development of the nursing procedure to promote patient's sleep, so the nurses' perspective on the questionnaire has been taken into account. The data were collected through self‐assessment by the nursing staff, which can be viewed critically. While this study focused on describing sleep‐promoting interventions from the nurses' perspective, patients may experience the studied phenomenon differently. Since patient viewpoints were not explored, the nurses' self‐assessments may reflect the prevailing ward culture rather than the actual needs of the patients. The results might have been different if we had assessed how nurses operate in the ward in general.

Despite the reminder sent during the data collection, we were unable to get more respondents to participate. However, the total response rate was 27%, which can be considered moderate in an electronic survey. Both quantitative and qualitative methods were used in the study, and the results can be considered to support each other and together give a wider perspective on the theme. The study was conducted in two Finnish hospitals, which may affect the extent to which the results are available. However, the study has produced an image of how nurses implement interventions that support patients' sleep.

## 5 Conclusions

Nurses assess that they are implementing patient sleep‐promotion interventions well. Nurses realise patients' sleep promotion as a part of their daily activities. However, nurses may lack skills and knowledge regarding sleep promotion. Due to shorter treatment periods, patient guidance on the importance of sleep for recovery from illness is emphasised, and this requires expertise from the nurses. Small interventions, such as providing earplugs and eye masks, can be used to promote patient's sleep, but it is also necessary to support the skills of nurses and implement jointly agreed practices. Nurse managers have an important role in leading evidence‐based practice in sleep promotion as well. Nurse managers should enable professional development and the strengthening of competence so that the patients' fundamental care is carried out with high quality and nurses can perceive promoting patient's sleep as a part of good fundamental care.

## Author Contributions

Miia Vasse: conceptualization, methodology, investigation, formal analysis, writing – original draft and editing. Elina Paala: investigation, formal analysis, writing – review and editing. Mari Kangasniemi: methodology, investigation, formal analysis, writing – review and editing. Marita Koivunen: conceptualization, methodology, investigation, formal analysis, writing – review and editing. All authors reviewed and approved the final version of the manuscript.

## Funding

The study was financially supported by The Satakunta Hospital District, Finland; Government Research Financing, Decisions 301/2021, 128/2022; Wellbeing Services County of Satakunta 169/2023.

## Ethics Statement

This study received ethical approval from the ethics committee of the Higher Education Institutions in Satakunta, Finland (Decision 10. January 2022). The permission to conduct the study was obtained from the hospital authorities of the Satakunta Hospital District, Finland (Decision 129/22, 2. February 2022) and the Hospital District of Central Finland (Decision 7. February 2022). The questionnaire was accompanied by an introductory letter with information about the aim of the study and assurance of anonymity and confidentiality. Responding to the survey was considered informed consent to participate in the research.

## Conflicts of Interest

The authors declare no conflicts of interest.

## Data Availability

The data that support the findings of this study are available from the corresponding author upon reasonable request.
